# The higher the better? Defining the optimal beta-lactam target for critically ill patients to reach infection resolution and improve outcome

**DOI:** 10.1186/s40560-020-00504-w

**Published:** 2020-11-23

**Authors:** Christina Scharf, Uwe Liebchen, Michael Paal, Max Taubert, Michael Vogeser, Michael Irlbeck, Michael Zoller, Ines Schroeder

**Affiliations:** 1 Department of Anaesthesiology, University Hospital, LMU Munich, Marchioninistrasse 15, 81377 Munich, Germany; 2 Institute of Laboratory Medicine, University Hospital, LMU Munich, Munich, Germany; 3 Department I of Pharmacology, Centre for Pharmacology, Faculty of Medicine and University Hospital Cologne, University of Cologne, Cologne, Germany

**Keywords:** Meropenem, Piperacillin-tazobactam, Infection resolution, Mortality, Critical illness, Breakpoint of target attainment

## Abstract

**Objectives:**

Beta-lactam antibiotics are often subject to therapeutic drug monitoring, but breakpoints of target attainment are mostly based on expert opinions. Studies that show a correlation between target attainment and infection resolution are missing. This analysis investigated whether there is a difference in infection resolution based on two breakpoints of target attainment.

**Methods:**

An outcome group out of 1392 critically ill patients treated with meropenem or piperacillin-tazobactam was formed due to different selection criteria. Afterwards, three groups were created: group 1=free drug concentration (*f*) was < 100% of the time (*T*) above the minimal inhibitory concentration (MIC) (< 100% fT* >*_*MIC*_), group 2=100% fT* >*_*MIC*_ *<*_*4xMIC*_, and group 3=100% fT* >*_*4xMIC*_. Parameters for infection control, renal and liver function, and estimated and observed in-hospital mortality were compared between those groups. Statistical analysis was performed with one-way analysis of variance, Tukey post hoc test, *U* test, and bivariate logistic regression.

**Results:**

The outcome group consisted of 55 patients (groups 1–3, 17, 24, and 14 patients, respectively). Patients allocated to group 2 or 3 had a significantly faster reduction of the C-reactive protein in contrast to patients allocated to group 1 (*p* = 0.033 and *p* = 0.026)*.* Patients allocated to group 3 had a worse renal function, a higher Acute Physiology and Chronic Health Evaluation (APACHE II) score, were older, and had a significantly higher in-hospital mortality compared to group 1 (*p* = 0.017) and group 2 (*p* = 0.001). The higher mortality was significantly influenced by worse liver function, higher APACHE II, and higher Sequential Organ Failure Assessment (SOFA) score and norepinephrine therapy.

**Conclusion:**

Achieving the target 100% fT* >*_*MIC*_ leads to faster infection resolution in the critically ill. However, there was no benefit for patients who reached the highest target of 100% fT* >*_*4xMIC*_, although the mortality rate was higher possibly due to confounding effects. In conclusion, we recommend the target 100% fT* >*_*MIC*_ *<*_*4xMIC*_ for critically ill patients.

**Trial registration:**

NCT03985605

**Supplementary Information:**

The online version contains supplementary material available at 10.1186/s40560-020-00504-w.

## Introduction

The clinical pictures of sepsis and septic shock are associated with a high mortality and morbidity especially in critically ill patients [1, 2]. Efforts towards the optimization of therapy only led to a slightly better outcome in those patients in the last 10 years [3, 4]. In general, effective antibiotic therapy consists of the correct substance and therapeutic drug concentration [5, 6].

Beta-lactam antibiotics like meropenem and piperacillin-tazobactam are among the most used antibiotics in intensive care unit (ICU) patients and have broad-spectrum efficacy. However, especially in ICU patients, beta-lactam antibiotics show a huge pharmacokinetic variability [7–10]. In particular, hypoalbuminemia, capillary leakage, and organ replacement therapy are known to condition a high inter- and intraindividual variability in plasma concentration [11–13]. To optimize antibiotic therapy, therapeutic drug monitoring (TDM) is a common method; many data support its usage in clinical routines [13–15].

Basically, there are no generally accepted breakpoints as they are usually defined on the basis of expert opinions, rather than on clinical outcome parameters [16]. Even though a free drug concentration (*f*) of 40% of the time (*T*) above the minimal inhibitory concentration (MIC) (40% *f T >*_*MIC*_) is associated with adequate antimicrobial activity for beta-lactams in vitro [17], recent studies in ICU patients propose a target of 100% fT* >*_*MIC*_ for the critically ill [13, 18]. Moreover, many authors use an even higher breakpoint of 100% fT* >*_*4-10xMIC*_ [8, 9, 13, 19], probably due to the fact that the concentration in the effect compartment is lower than the blood concentration, and the efficacy might be better with higher antibiotic concentrations.

However, all these studies just show how targets can be better achieved for example with pharmacokinetic models but do not show an effect when the target had been gained [20, 21]. Furthermore, the ideal balance between infection resolution and the prevention of toxic side effects like nephrotoxicity and neurotoxicity is not known at the moment [22, 23].

Recently, Richter et al. showed the lowest mortality in patients treated with piperacillin and target attainment of 100% fT* >*_*MIC*_ *<*_*4xMIC*_ and the highest mortality in patients with target attainment of 100% fT* >*_*4xMIC*_ [24]. Furthermore, Dhaese et al. showed the lowest survival in patients treated with meropenem or piperacillin and target attainment of 100% fT* >*_*4xMIC*_ [25]. These data raise the question why patients with high antibiotic levels have a higher mortality rate and what target should be aimed instead. Therefore, a retrospective analysis of a routine therapeutic drug-monitoring program for meropenem and piperacillin (after administration of piperacillin-tazobactam) was performed to find the best target for infection resolution and patients’ outcome.

## Materials and methods

### Study setting

The present study took place in two anaesthesiologically managed ICUs of the university hospital in Munich.

Clinical and laboratory parameters including meropenem and piperacillin serum concentrations were documented between January 2018 and January 2020. The local institutional review board approved the study (registration number 18-578). Written consent was obtained from the patients or their legal representatives in line with the vote of the review board. The study was registered at clinicaltrials.gov (NCT03985605).

### Laboratory testing and data collection

Meropenem and piperacillin serum concentrations were measured with a published liquid chromatography tandem mass spectrometry (LC-MS/MS) method [26]. For demographic data evaluation, clinical variables and laboratory variables were collected from the laboratory information system.

### Study population and selection criteria

All patients who were treated with meropenem or piperacillin-tazobactam in the TDM program were screened. Indications of antibiotic therapy, dosage, dosing regimen, and dose adaption were at the discretion of the attending physicians. All antibiotic concentrations were trough level.

Exclusion criteria were:

- Less than three serum trough samples on consecutive days.
- No detection of a pathogen from 7 days before antibiotic therapy till the end of antibiotic therapy.
- The pathogen was not susceptible to meropenem or piperacillin-tazobactam.
- Therapy with another effective antibiotic.
- The pathogen was probably not the reason for the infection.
- Detection of other pathogens (especially fungal and viral pathogens).
- Surgical clean up.

### Pharmacokinetic/pharmacodynamic target

Two different breakpoints of target attainment were used (100% fT* >*_*MIC*_ and 100% *fT >*_*4xMIC*_) that led to three different groups (group 1 = < 100% fT* >*_*MIC*_, group 2 = 100% fT* >*_*MIC*_ *<*_*4xMIC*_, and group 3 = 100% fT* >*_*4xMIC*_). The period under review was three trough concentrations after achieving the steady state (36–48 h after the initiation of antibiotic therapy). Minimal inhibitory concentration was defined by the clinical sensible (S/I) breakpoint of the detected pathogen by the European Committee on Antimicrobial Susceptibility Testing (EUCAST).

### Statistical analysis

Statistical analysis was performed with IBM SPSS statistics (Version 26.0. IBM Corp., Armonk, NY, USA). The effect of target attainment on the reduction of C-reactive protein and interleukin-6, on the change in bilirubin, serum alanine aminotransferase, serum aspartate aminotransferase, glutamine-glutamyl transferase, creatinine, urea, creatinine clearance (CLCR), SOFA score, and APACHE II score was investigated. In addition, the number of patients with neurological disorders, renal replacement therapy, and hospital mortality was evaluated. Estimated mortality was calculated based on the APACHE II score on the first day of evaluation. The estimated mortality based on the APACHE II score was 15%, 25%, 40%, 55%, 75%, and 85% for the APACHE II score ranges 10–14, 15–19, 20–24, 25–29, 30–34, and > 34 points, respectively [27]. Furthermore, it was investigated whether the site of infection had a relevant influence on infection control and mortality. To determine the differences in the three groups, the initial characteristics on the first day of evaluation were used. To determine a significant difference, a one-sided analysis of variance (ANOVA) was performed combined with Tukey post hoc test. A bivariate logistic regression was performed to identify confounding factors that influenced the in-hospital mortality. A statistically significant difference was indicated if the *p* value was < 0.05. The Mann Whitney *U* test was performed to compare hospital mortality with estimated mortality.

## Results

### Demographic and clinical data

**Fig. 1:**
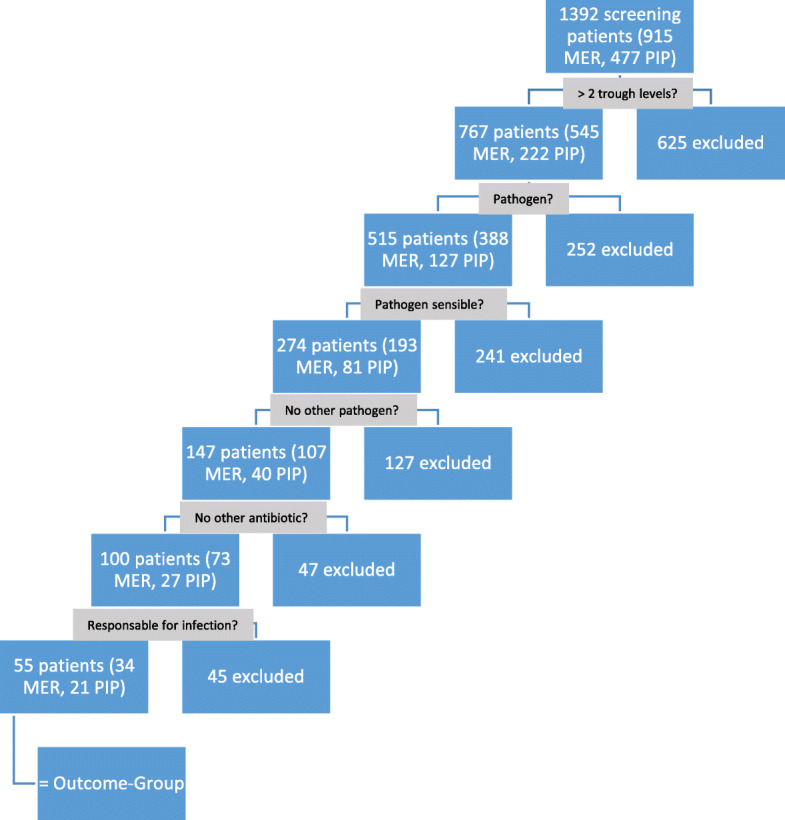
Selection criteria for detecting outcome patients. MER, meropenem; PIP, piperacillin

In total, 915 patients were treated with meropenem, and 477 patients were treated with piperacillin-tazobactam in the routine TDM program between January 2018 and January 2020. Those patients were screened for evaluation. The selection criteria are described in the “Materials and methods” section and can be seen in Fig. 1. A de-escalation of antibiotic therapy during the observation period did not occur in any of the patients evaluated.

Finally, 55 patients (34 patients treated with meropenem and 21 patients treated with piperacillin-tazobactam) fulfilled all the criteria and were defined as the “outcome group”. The “outcome group” formed the study population, which is shown below. The study population was divided into three groups as described in the “Materials and methods” section. The distribution in groups 1–3 based on target attainment was 17, 24, and 14 patients, respectively.

The reason for admission to the ICU was in descending order: organ transplantation (49.1%), acute respiratory distress syndrome (10.1%), sepsis of different origin (10.1%), polytrauma (9.1%), and uro-sepsis (5.5%). The place of infection or pathogen detection was in descending order: pulmonal (61.8%), abdominal (12.7%), urogenital (12.7%), blood (7.3%), and wound (5.5%). The median age of all patients was 62 years, and 60% were male. Most patients got a catecholamine therapy on day 1 of evaluation (81.8%) and needed invasive ventilation (74.5%). There were no relevant differences in patient characteristics in patients treated with meropenem or piperacillin-tazobactam. Further patient characteristics especially in the different groups can be found in Table 1.

**Table 1:** Patient characteristics in different groups

Parameter	All: ***n*** (%), median [min, max]	Group 1: ***n*** (%), median [min, max]	Group 2: ***n*** (%), median [min, max]	Group 3: ***n*** (%), median [min, max]
Age, years	62 [23, 90]	58 [23, 75]	62 [27, 84]	68 [48, 90]
Male/female	33/22	9/8	15/9	9/5
BMI, kg/m^2^	24.8 [15.2, 94.5]	24.0 [16.8, 94.5]	25.0 [20.4, 49.2]	26.0 [19.0, 30.1]
CRP d1 (mg/dL)	12.0 [0.7, 36.4]	9.8 [0.7, 32.5]	13.8 [1.1 28.2]	12.7 [0.7, 36.4]
ΔCRP (d1–d3, mg/dL)	3.0 [− 17.1, 18.2]	− 0.3 [− 17.1, 17.9]	4.5 [− 11.5, 12.8]	4.45 [− 6.0, 18.2]
IL-6 d1 (pg/mL)	93.1 [4, 2469]	101 [50.6, 470]	90.2 [5.1, 530]	108.2 [10.7, 2469]
ΔIL-6 (d1–d3, pg/dL)	48 [− 727, 2327]	50 [− 727, 433]	46.5 [− 138, 486]	84.7 [− 68.1, 2327]
APACHE II d1	25 [12, 51]	24 [16, 34]	25 [12, 37]	28.5 [18, 51]
ΔAPACHE–II (d1–d3)	4 [− 13, 22]	8 [− 6, 17]	3 [− 7, 22]	4 [− 13, 14]
SOFA d1	10 [3, 19]	9 [3, 14]	11 [5, 16]	11 [3, 19]
ΔSOFA (d1–d3)	4 [− 8, 9]	2 [− 8, 9]	2 [− 2, 7]	2 [− 4, 6]
Body temperature > 38.0 °C d1	17 (30.1)	4 (23.5)	9 (37.5)	4 (28.6)
Catecholamine therapy d1	45 (81.8)	12 (70.6)	20 (83.3)	13 (92.9)
Invasive ventilation d1	41 (74.5)	11 (64.7)	18 (75.0)	12 (85.7)
Bilirubin d1 (mg/dL)	0.9 [0.2, 11.9]	0.8 [0.2, 10]	0.9 [0.2, 4.9]	0.9 [0.4, 11.9]
ΔBilirubin (d1–d5, mg/dL)	0.2 [− 6.1, 8.7]	0.2 [− 1.5, 1.1]	0.2 [− 0.3, 2.3]	0.1 [− 6.1, 8.7]
AST d1 (U/L)	69.5 [16, 1359]	92 [52, 850]	64 [16, 1359]	61 [25, 693]
ΔAST (d1–d5, U/L)	25 [− 563, 1223]	47 [− 60, 792]	8 [− 563, 1223]	0 [− 168, 645]
ALT d1 (U/L)	29 [7, 2018]	28 [14, 987]	30 [8, 2018]	23 [7, 695]
ΔALT (d1–d5, U/L)	− 3 [− 322, 1662]	− 3 [− 40, 741]	− 5 [− 322, 1662]	1 [− 250, 415]
GGT d1 (U/L)	38 [11, 850]	28 [14, 547]	41 [11, 850]	97.5 [11, 618]
ΔGGT (d1–d5, U/L)	− 30 [− 1643, 457]	− 30 [− 522, 249]	− 29.5 [− 603, 101]	− 30.5 [− 1643, 457]
Creatinine d1 (mg/dL)	0.8 [0.4, 5.3]	0.7 [0.5, 1.2]	0.8 [0.6, 1.8]	1.2 [0.8, 5.3]
Δ creatinine (d1–d5, mg/dL)	0.05 [–3.4, 2]	0.0 [− 0.3, 2]	0.1 [–0.5, 0.7]	0.0 [–3.4, 0.4]
Urea d1 (mg/dL)	34 [18,131]	30.5 [18, 61]	34 [20, 131]	71 [29, 113]
Δ urea (d1–d5, mg/dL)	− 9.5 [− 134, 51]	− 9.5 [− 48, 9]	− 7 [− 134, 51]	− 23 [− 76, 27]
CLCR d1 (mL/min)	79 [4, 200]	146.5 [59, 200]	76 [13, 170]	42 [4, 80]
Δ CLCR (d1–d5, mL/min)	− 17 [− 139, 78]	5.5 [− 139, 78]	− 24.5 [− 116, 70]	− 10 [− 41, 3]
RRT	9 (16.4)	1 (5.9)	3 (12.5)	5 (35.7)
ECMO d1	2 (3.6)	0 (0.0)	1 (4.2)	1 (7.1)
In-hospital mortality	13 (23.6)	3 (17.6)	2 (8.3)	8 (57.1)
Trough-level meropenem (mg/L)	5.0 [0.25, 65.3]	1.85 [0.25, 20.4]	5.4 [2.17, 27.4]	18.3 [8.48, 65.3]
Trough-level piperacillin (mg/L)	40.1 [0.5, 400]	8.09 [0.5, 27.2]	25 [7.2, 156]	89.2 [46, 400]

*CRP* C-reactive protein, *d1* day 1 of evaluation, *IL-6* interleukin-6, *AST* serum aspartate aminotransferase, *ALT* serum alanine aminotransferase, *GGT* glutamine-glutamyl transferase, *CLCR* creatinine-clearance, *RRT* renal replacement therapy, *ECMO* extracorporeal membrane oxygenation, *d1–d5* day 1–day 5

Antibiotic trough concentrations of every patient can be found in Figure S1 (supplemental file). The detected pathogens and minimal inhibitory concentrations of every patient can be found in Table S1 (supplemental file).

### Baseline characteristics in the different groups

Results of the statistical analysis of the baseline characteristics in the different groups can be found in Table 2. One-way ANOVA showed significant differences in creatinine, urea, CLCR, age, and APACHE II score on the first day of evaluation. Patients allocated to group 3 had a significant higher creatinine and urea levels compared to group 1 (*p* < 0.001, *p* = 0.001) and group 2 (*p* < 0.001, *p* = 0.021). Furthermore, CLCR was significantly lower in patients allocated to group 3 compared to group 1 (*p* < 0.001) and group 2 (*p* = 0.007) as well as lower in group 2 compared to group 1 (*p* = 0.002). Moreover, APACHE II score was significantly higher in patients allocated to group 3 compared to group 1 (*p* = 0.03) and with a tendency compared to group 2 (*p* = 0.061). Patients allocated to group 3 were significantly older than patients allocated to group 1 (*p* = 0.007).

**Table 2:** Statistical analysis of the patient characteristics with one-way analysis of variance and post hoc Tukey test

	One-way ANOVA (*p*)	Post hoc 1–2 (*p*)	Post hoc 1–3 (*p*)	Post hoc 2–3 (*p*)	95% CI 1–2	95% CI 1–3	95% CI 2–3
**Baseline parameters**							
IL 6 d1	0.286	0.999	0.374	0.304	− 254–263	− 458–129	− 443–105
CRP d1	0.805	0.861	0.813	0.985	− 8.08–5.19	− 9.47–5.62	− 7.52–6.54
Creatinine d1	**< 0.001**	0.702	**< 0.001**	**< 0.001**	− 0.7–0.4	− **1.7 to** − **0.5**	− **1.5 to** − **0.3**
Urea d1	**0.001**	0.260	**0.001**	**0.021**	− 35–7	− **64 to** − **16**	− **48 to** − **3**
CLCR d1	**< 0.001**	**0.002**	**< 0.001**	**0.007**	**17–88**	**61–138**	**11–83**
RRT d1	0.066	0.832	0.066	0.146	− 0.3–0.2	− 0.6–0.02	− 0.5–0.06
Bilirubin d1	0.499	0.947	0.677	0.475	− 1.5–1.9	− 2.6–1.2	− 2.6–0.9
ALT d1	0.723	0.781	0.754	0.988	− 174–306	− 196–361	− 249–281
AST d1	0.728	0.938	0.900	0.706	− 308–230	− 250–361	− 191–379
GGT d1	0.283	0.931	0.482	0.261	− 108–145	− 213–75	− 222–46
APACHE II d1	**0.026**	0.989	0.061	**0.03**	− 5.0–5.6	− 11.8–0.2	− 11.7 to − 0.5
SOFA d1	0.133	0.123	0.338	0.703	− 4.3–1.1	− 5.7–0.5	− 3.8–1.9
Age	**0.010**	0.204	**0.007**	0.183	− 17.1–2.8	− 26.3 to − 3.6	− 18.4–2.7
BMI	0.845	1.00	0.874	1.00	− 11.0–11.0	− 10.0–15.1	− 9.1–14.3
Sex	0.78	0.82	0.806	0.994	− 0.5–0.3	− 0.6–0.3	− 0.4–0.4
**Delta parameters**							
ΔIL-6 (d1–d3)	0.236	0.959	0.255	0.319	− 291–231	− 494–100	− 444–109
ΔCRP (d1–d3)	**0.015**	**0.033**	**0.026**	0.903	− **11.2 to** − **0.4**	− **12.9 to** − **0.67**	− 6.7–4.7
ΔCreatinine (d1–d5)	0.253	0.900	0.236	0.37	− 0.4–0.6	− 0.2–1.1	− 0.3–1.0
ΔUrea (d1–d5)	0.327	0.79	0.657	0.298	− 34–20	− 21–45	− 12–50
ΔCLCR (d1–d5)	0.419	0.398	0.891	0.763	− 22–73	− 45–66	− 67–37
ΔBilirubin (d1–d5)	0.946	0.957	1.00	0.961	− 1.3–1.7	− 1.7–1.7	− 1.7–1.4
ΔALT (d1–d5)	0.526	0.632	0.553	0.978	− 174–306	− 196–361	− 249–281
ΔAST (d1–d5)	0.781	0.999	0.841	0.782	− 242–232	− 206–330	− 175–310
ΔGGT (d1–d5)	0.765	0.951	0.750	0.869	− 190–244	− 171–319	− 175–268
ΔAPACHE II (d1–d5)	0.227	0.358	0.231	0.887	− 1.3–4.7	− 1.0–5.6	− 2.4–3.6
ΔSOFA (d1–d5)	0.215	0.197	0.766	0.597	− 1.7–10.3	− 4.8–8.6	− 10.3–1.7
Neurological disorder d5	0.43	0.473	0.518	0.997	− 0.6–0.2	− 0.6–0.2	− 0.4–0.4
In-hospital mortality	**0.002**	0.728	**0.001**	**0.017**	− 0.2 – 0.4	− **0.7 to** − **0.06**	− **0.8 to** − **0.2**
Estimated mortality	0.123	0.996	0.198	0.135	− 0.1 – 0.1	− 0.3–0.05	− 0.3–0.03

*ANOVA* analysis of variance, *CI* confidence interval, *IL-6* interleukin-6, *CRP* C-reactive protein, *ALT* serum alanine aminotransferase, *AST* serum aspartate aminotransferase, *GGT* glutamine glutamyl transferase, *CLCR* creatinine clearance, *RRT* renal replacement therapy, *d* day, *BMI* body mass index

### Effect of antibiotic concentration on infection resolution

There was no significant difference in the change of interleukin-6 from day 1 to day 3 (∆interleukin-6) between the three different groups (*p* = 0.236). Detailed statistical results can be found in Table 2.

In contrast, there was a significant difference in the change of CRP from day 1 to day 3 (∆CRP) detected in the one-way ANOVA (*p* = 0.015). Post hoc Tukey test showed a significantly higher CRP decreased in patients allocated to group 2 (*p* = 0.033, 95% CI 11.2 to − 0.4) and group 3 (*p* = 0.026, 95% CI 12.9 to − 0.67) compared to group 1. However, there was no significant difference between groups 2 and 3 (*p* = 0.903).

**Fig. 2:**
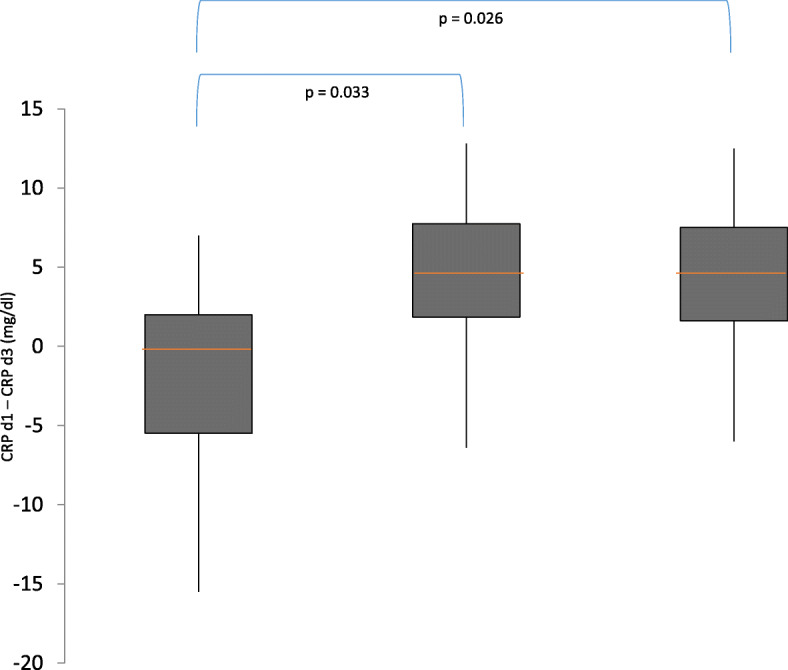
ΔCRP (day 1–day 3) in the three different groups. The red line represents the median, the gray boxes the interquartile range, and the whiskers the range of 1.5 times of the interquartile range. d, day, CRP, C-reactive protein

Figure 2 shows the change in the CRP (∆CRP) from day 1 to day 3 in the different groups using boxplots.

### Effect of antibiotic concentration on renal and liver function

There was no significant change in renal function from day 1 to day 5 (∆renal function parameters) in the different groups. Significant differences could neither be seen in creatinine (*p* = 0.253) or CLCR (*p* = 0.419) nor in urea (*p* = 0.327).

There was also no significant difference in the change of bilirubin from day 1 to day 5 (∆ bilirubin) between the different groups (*p* = 0.946). This is the same for alanine aminotransferase (∆ALT) (*p* = 0.526), aspartate aminotransferase (∆AST) (*p* = 0.781), and glutamine glutamyl transferase (∆GGT) (*p* = 0.761). Detailed information can be found in Table 2.

### Effect of antibiotic concentration on neurological disorder and ICU scores

There was no significant difference in the frequency of neurological disorders like inadequate waking reaction or delirium on the fifth day of evaluation in the different groups (*p* = 0.43). This was the same for the two common intensive care medicine scores. Neither the APACHE II score (*p* = 0.227) nor the SOFA score (*p* = 0.215) showed a significant difference between day 1 and day 5 (∆APACHE II/SOFA score) in the three groups. Detailed information can be found in Table 2.

### Effect of the infection focus on patients’ outcome

One-way ANOVA with post hoc Tukey test showed that the infection focus had no significant impact on clinical and laboratory patient characteristics. The influence of the infection focus was examined for the parameters: APACHE II score day 1, SOFA score day 1, CRP day 1, CRP day 1–day 3, IL-6 day 1, bilirubin day 1, AST day 1, creatinine day 1, need of renal replacement therapy day 1, age, sex, body mass index (BMI), need of invasive ventilation day 1, norepinephrine > 1.0 mg/h day 1, in-hospital mortality, estimated mortality based on APACHE II score day 1, and group.

### Differences in in-hospital mortality

One-way ANOVA showed a significant difference in in-hospital mortality in the different groups (*p* = 0.002). Post hoc Tukey test showed a significantly higher in-hospital mortality in patients allocated to group 3 compared to group 1 (*p* = 0.001, CI − 0.7 to − 0.06) and group 2 (*p* = 0.017, CI − 0.8 to − 0.2). In contrast, there was no significant difference between groups 1 and 2 (*p* = 0.728).

**Fig. 3:**
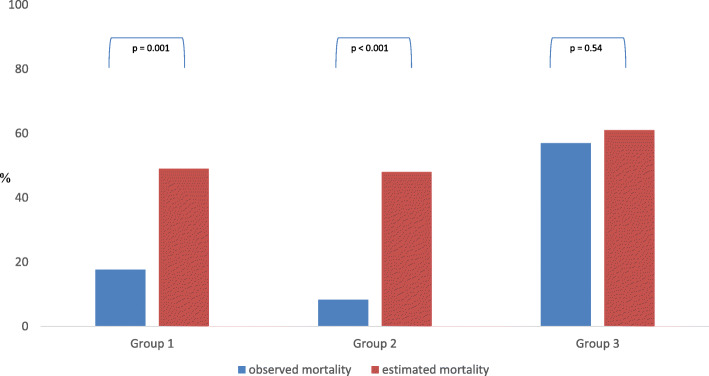
Observed and estimated mortality in the different groups and statistical results of the *U* test

Observed mortality in patients allocated to groups 1, 2, and 3 was 17.6%, 8.3%, and 57.0%, respectively. In contrast, estimated mortality based on the APACHE II score on the first day of evaluation was 49.0%, 48.0%, and 61.0%, respectively. There was a significantly lower observed than estimated mortality in patients allocated to group 1 (*p* = 0.001) and group 2 (*p* < 0.001) detected with the *U* test. No statistical difference can be found in patients allocated to group 3 (*p* = 0.54). Furthermore, there was no statistically significant difference (*p* = 0.376) in the observed mortality rate (assuming an identical predicted mortality), although a numerical difference (17.6% vs 8.3% in groups 1 and 2) can be seen. Figure 3 shows the relationship between the observed and the estimated mortality in the different groups and the statistical results of the *U* test.

### Confounder that affect in-hospital mortality

In order to be able to distinguish if higher in-hospital mortality is based on antibiotic concentration or on confounding factors, a bivariate logistic regression was performed with in-hospital mortality as the dependent variable. The model was created once with and once without the independent variable “group”. Other independent variables were APACHE II score day 1, SOFA score day 1, CRP day 1, IL-6 day 1, bilirubin day 1, AST day 1, creatinine day 1, need of renal replacement therapy day 1, age, sex, body mass index (BMI), need of invasive ventilation day 1, norepinephrine > 1.0 mg/h day 1, infection focus, and estimated mortality based on APACHE II score day 1.

Logistic regression without “group” as independent variable showed a significant result (*p* = 0.02) in the omnibus test of the model coefficients. The regression analysis showed that APACHE II score day 1 (*p* = 0.03, CI 1.08, 4.59), SOFA score day 1 (*p* = 0.05, CI 0.37, 1.00), bilirubin day 1 (*p* = 0.014, CI 1.3, 10.67), AST day 1 (*p* = 0.015, CI 0.96, 1.00), norepinephrine > 1.0 mg/h day 1 (*p* = 0.023, CI 1.68, 1099), and estimated mortality based on APACHE II score day 1 (*p* = 0.031, CI 0.00, 0.095) were significant confounder on in-hospital mortality. In contrast, renal function (creatinine day 1 and CRRT) or infection focus had no significant impact on in-hospital mortality.

Logistic regression with “group” as independent variable showed on the one hand that there were no changes in the known confounders and on the other hand that “group” is no significant confounding factor on in-hospital mortality (*p* = 0.38).

The repetition of the logistic regression with the subgroup (group 2 + 3) and the subgroup (group 1 + 2) showed no significant model and no significant confounding factors.

## Discussion

The reason for the lack of outcome data in septic patients treated with therapeutic drug-monitoring controlled antibiotic therapy might be that a “septic patient” is a too heterogeneous group [28]. Even if the therapy of sepsis is complex and multifactorial, the appropriate target for beta-lactams (as some of the most often used antibiotics) is crucial [29] but not known at the moment [16, 30, 31]. Most recommendations are based on animal models or experimental studies or refer to pathophysiological considerations [17, 32–36]. Our new approach was forming an outcome group that the effect of antibiotic therapy is not masked by other measures and influences. A similar approach was recently performed by Tannous et al. to predict the outcome of patients infected with *Pseudomonas aeruginosa* and treated with piperacillin-tazobactam [37].

However, there are only a few studies addressing the optimal target based on parameters of infection resolution and outcome. Roberts et al. showed a faster reduction of the APACHE II score in ICU patients targeting 100% fT* >*_*MIC*_ [16]. These findings were recently confirmed in a large study including more than 480 patients [24]. Furthermore, it had been confirmed that 100% fT* >*_*MIC*_ was superior to lower targets for cefepime or ceftazidime [32, 33]. Still, many recommendations and studies aim for targets as high as *f T >*_*4-8xMIC*_ [13, 38] without clear evidence.

CRP is one of the most important outcome parameters in septic patients [39, 40]. It is well known that a reduction in CRP in septic patients treated with antibiotics led to lower mortality compared to patients with no reduction of CRP [41]. Therefore, it is one of the best prognostic parameters to evaluate whether antibiotic therapy is helpful [42]. To the best of our knowledge, we are the first researchers to show a statistically significant faster reduction of CRP in patients with target attainment of 100% fT* >*_*MIC*_ in contrast to the lower target.

It remains to be discussed why target attainment did not lead to a significantly faster reduction of interleukin-6, although it is an important parameter to evaluate the success of antibiotic therapy in patients diagnosed with sepsis [43, 44]. One explanation might be that two-thirds of the patients got hydrocortisone or high-dose prednisolone, in line with the common concept of sepsis therapy or after solid organ transplantation [45, 46]. This leads to a suppression of cytokine expression and thus to falsified IL-6 values.

Different authors described neurotoxicity as a toxic side effect of high beta-lactam concentrations [23, 47–50]. This cannot be seen in our evaluation, probably due to a too small study population and the fact that most of the patients were intubated and got medication for sedation. Furthermore, also nephro- or hepatotoxic effects cannot be seen in our study population. Even though toxic side effects of beta-lactam antibiotics were rather rare and did not occur in our population, questions remain open about the benefit of even higher trough levels and the economic viability with regard to the higher consumption of antibiotics. In addition, it must be noted that just because a dosage regimen without benefits had no measurable side effects, it is still not useful.

Looking at the different groups, the kidney function was contrary to the achieved target (the worse the kidney function, the higher the target). Thus, it is possible to record that the high beta-lactam concentration was most likely induced by impaired renal function, as described before [9]. Moreover, patients who achieved the highest target were older and had a significantly higher APACHE II score on the first day of evaluation, which is one of the best predictor for the outcome of ICU patients [51]. In summary, the patients allocated to group 3 were thus older and sicker. This is already an indication that the higher mortality in patients allocated to group 3 was caused by confounders and not by the antibiotic therapy. This issue is therefore interesting, as the higher mortality in patients with target attainment of 100% fT* >*_*4xMIC*_ had already been described but never clearly explained [24, 25].

To further explore this question, a logistic regression analysis with the in-hospital mortality as dependent variable was performed. Logistic regression of all patients showed that APACHE II and SOFA score, bilirubin and AST, norepinephrine > 1.0 mg/h, and estimated mortality were relevant confounders on in-hospital mortality. On the contrary, patients’ kidney function, the focus of the infection, and the variable “group” were no confounder on the mortality. Furthermore, the infection site itself had no influence on mortality or laboratory parameters in the analysis of variance. However, these results must be interpreted with caution in view of the partly small number of patients.

Nevertheless, the question remains open, why the observed mortality in patients allocated to groups 1 and 2 was significantly lower than the estimated mortality, whereas no difference can be seen in patients allocated to group 3. Furthermore, the question why the observed mortality of patients allocated to group 2 was ultimately less than half as high compared to group 1 (8.3% versus 17.6%) with identical estimated mortality rates (49% and 48%) cannot be answered with our study results. Hypothetically, this difference was caused by reaching the target 100% fT* >*_*MIC*_ *<*_*4xMIC*_. To prove this hypothesis, a study with a larger number of patients seems reasonable.

Our study has several limitations. It is possible that eligible patients were eliminated because the causal pathogen was not detected in microbiological testing. In contrast, it is remarkable that 55 patients were enough to show a superiority in CRP decline when the target 100% fT* >*_*MIC*_ was attained. Furthermore, we used the MIC supported by the EUCAST and not the real MIC of the detected pathogen. This is in line with our clinical standard. However, MICs determined by the microbiology laboratory might be superior to the EUCAST data. Moreover, the dose recommendation refers to critically ill patients with pathogen detection that is susceptible to beta-lactams. A transfer to other antibiotic classes is not possible. Furthermore, the observation period of 3 days after achieving the steady state might be too short to establish a relationship to the factor “in-hospital mortality”. The exact threshold at which the in-hospital mortality started to increase in patients allocated to group 3 most likely due to confounding effects can also not be answered by our results and requires further studies in the future. Last, data evaluation was in a retrospective setting. Therefore, while this study was a hypotheses-forming one, it did not provide conclusive evidence for the optimal target.

## Conclusions

Forming an outcome group is an innovative approach to find an appropriate target for infection resolution and better outcome in patients treated with beta-lactam antibiotics. Our results suggest that the target 100% fT* >*_*MIC*_ is eligible to reach infection resolution in the critically ill.

However, achieving the target 100% fT* >*_*4xMIC*_ was associated with higher mortality probably due to confounding effects like higher APACHE II score and higher age. Furthermore, there was no benefit such as better infection resolution compared to patients, who achieved the target 100% fT* >*_*MIC*_ *<*_*4xMIC*_.

Finally, we recommend 100% fT* >*_*MIC*_ *<*_*4xMIC*_ as the optimal beta-lactam target for the critically ill.

## Supplementary Information

**Additional file 1:** **Figure S1.** Trough concentrations (mg/L) of all outcome patients. **Table S1**. Detected pathogens of all outcome patients.

## Data Availability

All data generated during this study are included in this article.

## Abbreviations

ALT: Serum alanine aminotransferase
ANOVA: Analysis of variance
APACHE: Acute Physiology and Chronic Health Evaluation
AST: Serum aspartate aminotransferase
CI: Confidence interval
CLCR: Creatinine-clearance
CRP: C-reactive protein
EUCAST: European Committee on Antimicrobial Susceptibility Testing
*f*: Free drug concentration
GGT: Glutamine-glutamyl transferase
ICU: Intensive care unit
IL-6: Interleukin-6
LC-MS/MS: Liquid chromatography tandem mass spectrometry
MER: Meropenem
MIC: Minimal inhibitory concentration
PIP: Piperacillin
SOFA: Sequential Organ Failure Assessment
*T*: Time
TDM: Therapeutic drug monitoring
