# Supplementary material for: The higher the better? Defining the optimal beta-lactam target for critically ill patients to reach infection resolution and improve outcome
Source: J Intensive Care. 2020 Nov 23;8:86. doi: 10.1186/s40560-020-00504-w (PMC7686672; doi:10.1186/s40560-020-00504-w)
Supplement: Supplementary file 1 — Additional file 1: Figure S1. Trough concentrations (mg/L) of all outcome patients. Table S1. Detected pathogens of all outcome patients. [file 40560_2020_504_MOESM1_ESM.docx]

**Supplementary Materials**

**Figure S1.** Trough concentrations (mg/L) of all outcome patients.

**
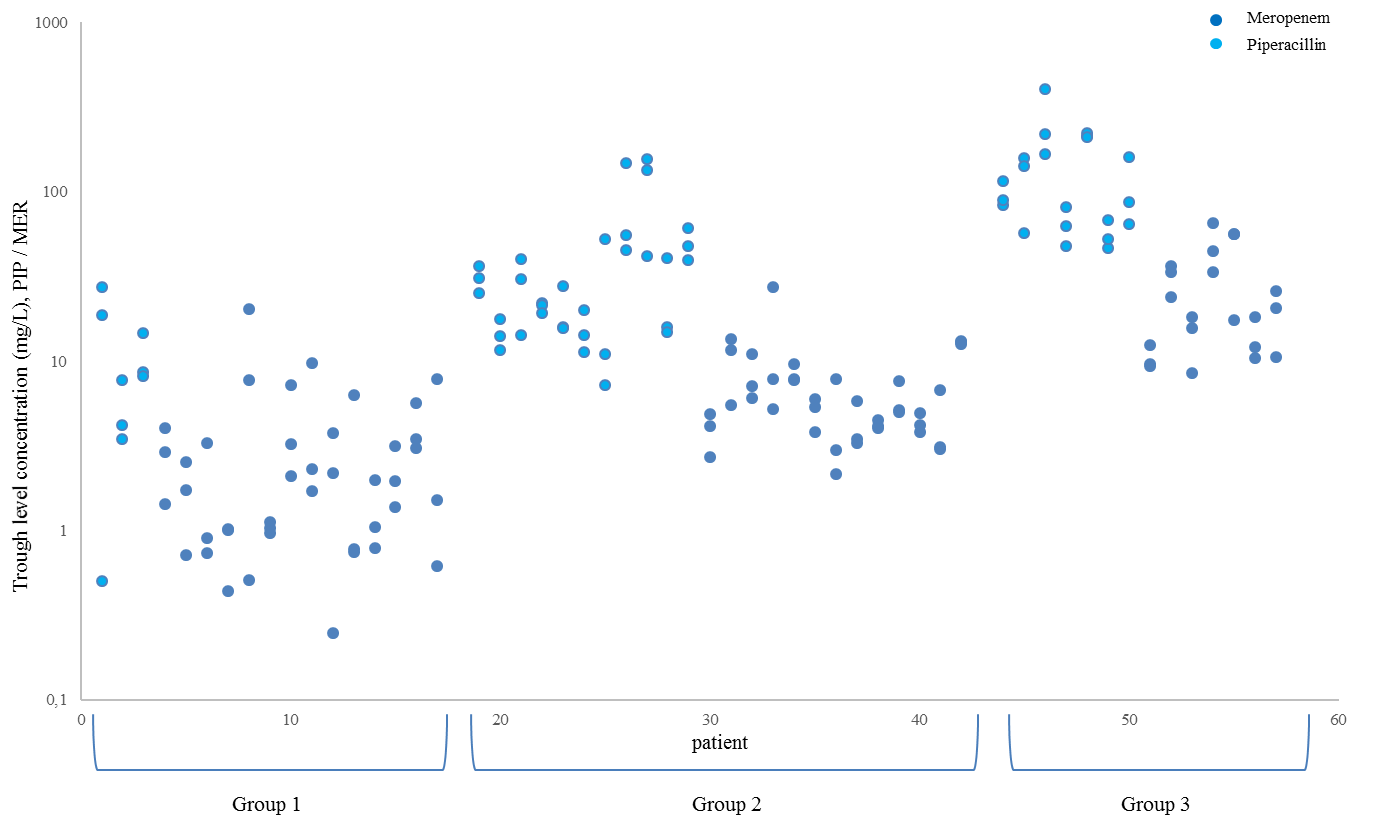
**

**Table S1.** Detected pathogens of all outcome patients.

| **Pathogen (MIC mg/L) PIP** | **Position (count)** |
| --- | --- |
| *Serratia marcescens* (8) | endotracheal extraction (1) |
| *Escherichia coli* (8) | endotracheal extraction (1), blood culture (3), abdominal (1) |
| *Klebsiella oxytoca* (8) | endotracheal extraction (1) |
| *Enterococcus faecalis/faecium* (4) | abdominal (1), urine (3), blood culture (1) |
| *Achromobacter xylosoxidans* (8) | bronchoalveolar lavage (1) |
| *Proteus mirabilis* (8) | urine (1), bronchoalveolar lavage (1) |
| *Pseudomonas aeruginosa* (16) | blood culture (1), endotracheal extraction (1), wound (2) |
| *Shewanella putrefaciens* (8) | endotracheal extraction (1) |
| *Streptococcus anginosus* (8) | endotracheal extraction (1) |
| **Pathogen (MIC mg/L) MER** | **Position (count)** |
| *Serratia marcescens* (2) | bronchoalveolar lavage (1), bronchus (2), liver (1), endotracheal extraction (2) |
| *Escherichia coli* (2) | bronchus (3), bronchoalveolar lavage (1), wound (2), urine (1), blood culture (1) |
| *Klebsiella oxytoca/pneumoniae* (2) | bronchus (2), endotracheal extraction (2) |
| *Enterococcus faecium* (8) | abdominal (3) |
| *Achromobacter xylosoxidans* (2) | bronchus (1) |
| *Proteus mirabilis* (2) | wound (1), urine (1) |
| *Pseudomonas aeruginosa* (2) | bronchoalveolar lavage (1), endotracheal extraction (1), bronchus (5) |
| *Acinetobacter junii* (2) | endotracheal extraction (1) |
| *Staphylococcus aureus* (4) | drainage (1) |
| *Streptococcus sanguinis* (2) | liver (1) |

Note: MIC: minimal inhibitory concentration, PIP: piperacillin, MER: meropenem,
